# Human immunodeficiency virus and multiple sclerosis: a review of the literature

**DOI:** 10.1186/s42466-019-0030-4

**Published:** 2019-08-20

**Authors:** Maria-Ioanna Stefanou, Markus Krumbholz, Ulf Ziemann, Markus C. Kowarik

**Affiliations:** Department of Neurology & Stroke, and Hertie Institute for Clinical Brain Research, Eberhard-Karls University of Tübingen, Hoppe-Seyler-Str. 3, 72076 Tübingen, Germany

**Keywords:** Human immunodeficiency virus, Multiple sclerosis, Antiretroviral therapy, Chemokine inhibitors, Acquired immune

## Abstract

Multiple sclerosis (MS) and human immunodeficiency virus (HIV) infection are frequent and well-studied nosological entities. Yet, comorbidity of MS and HIV has only been rarely reported in the medical literature. We conducted a literature search using the databases PubMed, Ovid and Google Scholar, with the aim of identifying published studies and reports concerning HIV and MS. Recent epidemiological studies indicated a negative association between MS and HIV in terms of a reduced risk of developing MS in HIV positive patients. Accumulating clinical evidence additionally suggests a possibly reduced relapse rate of MS in HIV patients. Nevertheless, it remains currently unclear whether this observed inverse correlation could be due to the HIV infection itself, HIV treatment or the combination of both. Among the limited cases of MS in HIV infected patients, MS occurrence was mainly reported during acute HIV infection or during HIV seroconversion. This finding is in line with reports of HIV-related autoimmune disorders, which also occur in early phases of HIV disease. Beneficial effects of antiretroviral therapy on MS activity were reported in few clinical cases. Yet, the single phase II clinical trial (INSPIRE), which investigated the effects of antiretroviral medication (using the integrase inhibitor raltegravir) in patients with relapsing-remitting MS, failed to corroborate any beneficial effects at group level. Nevertheless, recently published experimental evidence suggests that HIV treatments may hold therapeutic potential for MS treatment. Thus, further studies are warranted to firstly, delineate the immunological mechanisms underlying possible efficacy of HIV treatments in MS, and to secondly, assess whether repurposing of HIV drugs for MS could be a worthwhile future research objective.

## Background

**Fig. 1:**
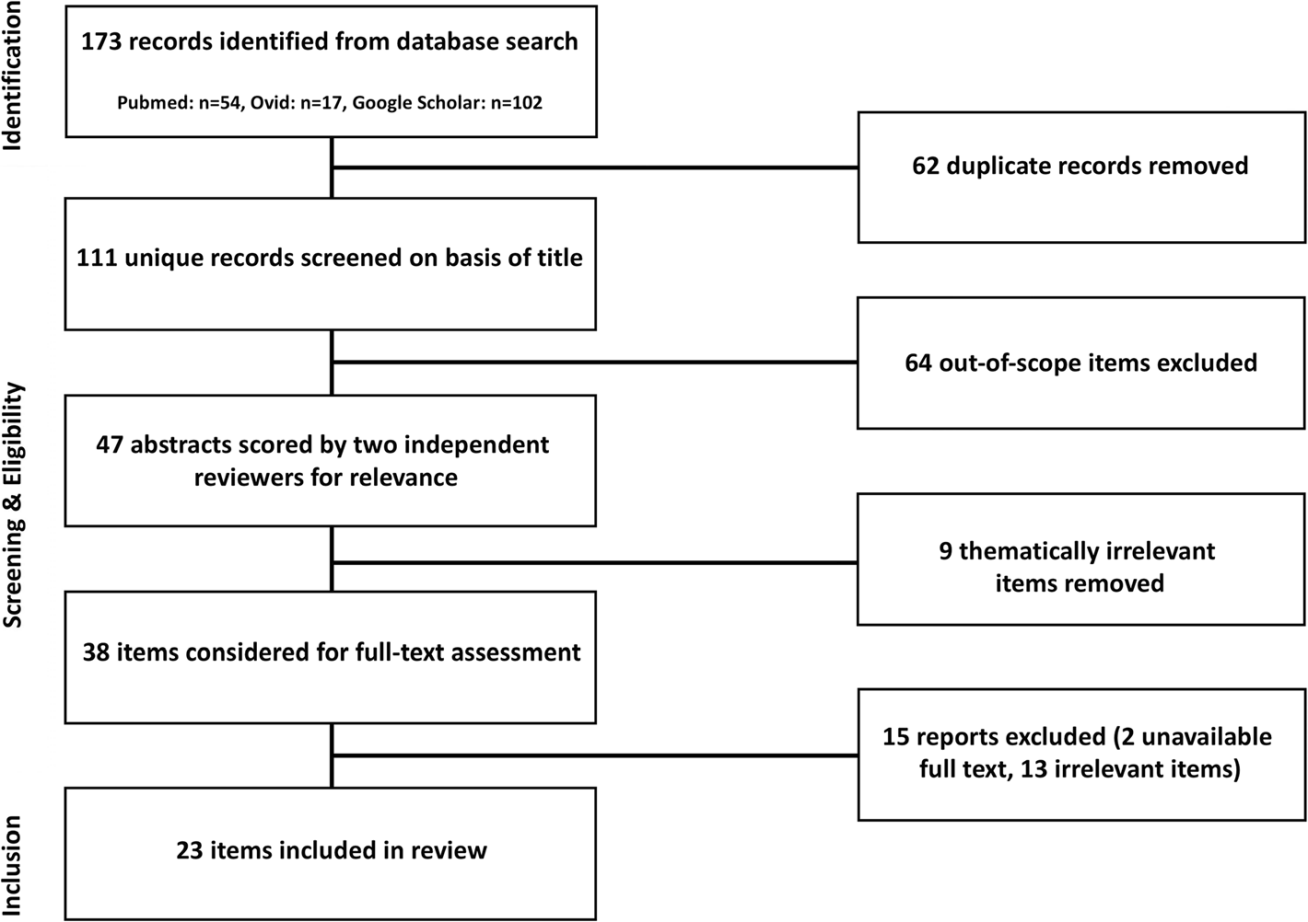
Flow chart of literature review process. Literature search was conducted using the databases PubMed, Ovid and Google Scholar, and search terms multiple sclerosis and human immunodeficiency virus or HIV or AIDS or antiretroviral therapy, to identify articles written in English or German, between 1985 and 2019. Two independent reviewers carried out the selection of the studies

Human immunodeficiency virus (HIV) infection is characterized by a progressive loss of CD4+ T lymphocytes, which leads to failure of the immune system and (if left untreated) to acquired immunodeficiency syndrome (AIDS). In contrast, CD4+ T cells (Th1/Th17 phenotype) are considered to play an important role in the presumably autoimmune pathogenesis of multiple sclerosis (MS) in orchestration with CD8+ T cells, B cells and cells of the innate immune system [27]. HIV infection not only leads to reduced CD4+ T cell numbers and an inverse CD4+/CD8+ ratio in the peripheral blood, but also shows similar effects on cerebrospinal fluid (CSF) T cells [50]. MS treatments like natalizumab, and less pronounced fingolimod, also lead to a reduced CD4+/CD8+ ratio in the CSF, similar to HIV patients [28, 46]. In both, natalizumab treated patients as well as HIV patients, John Cunnigham (JC) virus reactivation in the CNS often leads to progressive multifocal leukoencephalopathy (PML), indicating that the impact on the immune system with an inverse CSF CD4+/CD8+ ratio might have similar effects on certain opportunistic infections. Vice versa, it could be hypothesized, that the impaired immune system in HIV patients could result into less autoimmunity, a lower prevalence of MS or less disease activity. However, manifold data on HIV and MS rather highlight the complexity of immune responses and a differential role of certain immune cell subsets. Moreover, HIV infection is known to cause an immune dysregulation in certain disease states or under highly active antiretroviral therapy (HAART), which has been linked to development of other autoimmune and systemic diseases [49]. The aim of this paper is to review the current literature on HIV and multiple sclerosis, analyze recent findings from studies on HIV drugs in MS treatment and discuss future research directions. Literature search was conducted using the databases PubMed, Ovid and Google Scholar, and search terms multiple sclerosis and human immunodeficiency virus or HIV or AIDS or antiretroviral therapy, to identify articles written in English or German, between 1985 and 2019. Two independent reviewers carried out the selection of the studies (Fig. 1).

## HIV infection and risk for autoimmunity

Besides the ensuing CD4+ T-cell reduction, HIV infection has been shown to alter the mechanisms of immunological tolerance and autoimmunity in HIV-positive patients [41]. Current research has disproven the previously widely held belief that HIV infection generally suppresses autoimmunity [52], with recent evidence indicating a frequency of rheumatological syndromes in up to 60% of HIV-infected patients [49, 52]. During stage I of HIV disease (following the acute viral infection and while the immune system is still intact) [51], and during the phase of immune reconstitution following HAART, a wide spectrum of autoimmune diseases, including systemic lupus erythematosus, sarcoidosis, Guillain–Barré syndrome (GBS), immune thrombocytopenic purpura, polymyositis, Graves’ disease, myasthenia gravis (MG) and rheumatoid arthritis, have been reported [49]. Among these autoimmune disorders, some even appear more frequently in the HIV positive compared to general population, i.e., with reported frequencies for sarcoidosis, GBS, and myositis of 0.08% (for each disease) in HIV patients, compared to international prevalence estimated at 0.0125% for sarcoidosis, 0.0035% for GBS, and 0.0051% for myositis [49]. During stage II (while declined CD4+ cell counts and immunosuppression are noted), autoimmune disorders are not typically found [52]. During stage III and rarely during stage IV (while low CD4+ counts and AIDS are noted, respectively) [51], CD8+ T-cell induced autoimmune disorders such as psoriasis and diffuse lymphocytic syndrome may occur [52].

Autoimmune disorders are, thus, being increasingly acknowledged as significant comorbidities during the early HIV-stages and after HAART induction [49]. Studies focusing on the pathways of autoimmunity in HIV-infected individuals have suggested possible mechanisms that account for an autoimmune diathesis in affected patients, including: a) molecular mimicry processes [10], b) HIV-induced release of cytokines causing CD4+/polyclonal B-cell activation [49], c) emergence of autoantibodies [36], d) loss of regulatory CD8+ T-cells [36], and e) overt immune reconstitution after HAART induction [49]. In HIV-associated autoimmune peripheral neurologic disorders, including GBS and MG, similar inflammatory cascades in temporal association with the primary HIV infection, the period of seroconversion or within the first months after HAART have been hypothesized [5, 9]. Although the exact mechanisms for neurological autoimmunity during HIV infection are only partially understood, pathophysiological processes involving a) molecular mimicry through action of HIV-1 on neurons by neurotropic strains, and b) production of antibodies against myelin have been proposed [11].

## HIV infection and multiple sclerosis

In 2013, the first epidemiological report of negative association between HIV infection and multiple sclerosis (MS) was published from a Danish research group [37]. The researchers investigated 5018 first-diagnosed HIV patients and 50,149 healthy controls (matched for age and sex and followed-up for 31,875 and 393,871 person-years, respectively), using the Danish National Registry of patients (between 1994 and 2011) and the Danish MS Registry [6]. They found an incidence rate ratio (IRR) of 0.3 (95% CI 0.04 to 2.20) of MS among HIV patients compared to healthy controls [37]. While in this cohort the IRR did not reach statistical significance, a significant negative association between HIV and MS was subsequently confirmed in a large record linkage analysis published in 2015 [19]. Gold et al. investigated 21,207 HIV-positive patients and 5,298,496 controls (stratified for sex, age, region of residence, year of first hospital admission and socioeconomic status) and demonstrated a similar rate ratio (RR) of 0.38 (95% CI 0.15 to 0.79) of MS occurrence in HIV patients compared to controls [19].

Both these studies highlighted for the first time the inverse correlation between HIV and MS and raised salient questions regarding the possible etiologies of the noted lower risk for MS in the HIV-positive population. Firstly, a causal relationship between the HIV infection itself and the lower MS occurrence was postulated [19]. Secondly, a possible protective effect of HAART in the manifestation and/or course of MS was proposed [19]. However, no exact details on treatment time points and CD4+ T cells values were examined [19].

Looking at published cases, only a handful of reports of concomitant HIV infection and MS (MS-HIV) can be found in the literature [3, 7, 12, 14, 22, 23, 31]. Among these reports, only two clinical cases describe patients, diagnosed with MS according to the revised McDonald criteria [33, 39, 40], whose MS diagnosis significantly precedes HIV infection [32, 44], while in the rest of reports HIV precedes MS diagnosis. Compared with reports of HIV-related autoimmune disorders, the majority of published MS-HIV cases in the literature report a close temporal association of MS occurrence with the stages of acute HIV-1 infection or HIV seroconversion, but only rarely with the period following HAART induction [9]. To the best of our knowledge, only one published report describes a HIV-positive patient with a relatively suppressed CD4+ cell count, who developed clinical and radiographic MS worsening in the setting of HAART initiation [1]. Contrarily, several cases of MS have been reported in adult and pediatric patients during the early stages of HIV disease [3, 15, 23].

In 1989, Berger et al. first published a series of 7 patients with an “MS-like illness” occurring after HIV-infection [3], and in 1992, they reported a case of relapsing-remitting corticosteroid-responsive leukoencephalomyelopathy, clinically and histologically indistinguishable from MS in a patient with HIV-seroconversion [4]. After its first description, acute “MS-like disease” following HIV-infection was reported from several independent groups [17, 23, 24]. Coban et al. recently reviewed the clinical and imaging features of “MS-like disease” in HIV-patients and concluded that, although the clinical and imaging features are often indistinguishable from MS, atypical disease course (i.e., often with fulminating or rapid progression) and atypical cerebrospinal fluid findings (i.e., elevated protein levels or negative results of oligoclonal bands) are common [9]. This group also suggested a short latency between HIV viral acquisition and the development of MS symptoms in infected individuals [9]. Histological features of “MS-like disease” in the setting of HIV were presented from Graber et al., who showed pathological findings of extensive demyelination with reactive astrocytes, foamy macrophages and perivascular infiltrates with inflammatory cells, consistent with MS lesions in the phase of seroconversion in a HIV-positive patient [23].

Among the published reports of MS in HIV-positive patients, a recent account of MS according to the revised McDonald criteria [33, 39, 40] in a HIV-controller with normal CD4+ cell counts and a low viral load in the absence of HAART is of particular interest [8]. This report presented a patient diagnosed with relapsing-remitting MS after 5 years of non-progressive HIV-1 infection, indicating that firstly, chronic (besides early) HIV infection may equally precipitate MS, and that secondly, HIV infection per se probably holds no protective effect with respect to MS manifestation. Although a causal relationship between the HIV infection and the epidemiologically noted lower MS occurrence in the HIV-positive population cannot be ruled out [19, 37], the observations of persistent and enhanced immune responses during early and chronic HIV-infection [41, 42] have led some researchers to consider an even increased susceptibility to MS in HIV patients with predisposing genetic and environmental factors for MS [16, 41].

Another potential explanation for a possible underreporting of MS in HIV patients may lie in the current recommendations for MS diagnosis. Albeit most current MS diagnostic criteria, including the (revised) McDonald criteria [33, 39, 40, 47] do not specify the precise infectiological work-up required prior to MS diagnosis, diagnostic guidelines such as those of the German Neurological Society (DGN) [21], recommend that chronic infectious diseases (including HIV) should be excluded before MS diagnosis can be reached. Thus, it is plausible that the scarcity of MS-HIV cases, that are found in the published literature, may (at least partially) be attributed to the fact that MS diagnosis in HIV patients remains underreported or even underrecognized.

## The use of antiretroviral drugs in MS

A number of clinical cases have recently indicated that HIV-infected MS patients who received HAART had a less severe clinical course of MS, even in the absence of MS treatment [7, 12, 31]. This evidence could imply that if an inverse correlation between HIV and MS exists, this could be due to the effect of HAART on MS rather than due to the effects of HIV infection itself [34]. Therefore, several studies emerged over the past few years, examining the possible contribution of HAART in the course of MS in HIV-positive individuals, along with the possible contribution of HAART in the amelioration of overall risk for MS in the HIV-positive population [34].

To the best of our knowledge, a total of 6 clinical cases of patients (5 HIV-positive and 1 HIV-negative) (Table 1), who presented indefinite remission or resolution of MS symptoms after induction of HAART over long follow-up periods, have been reported in the literature [7, 13, 14, 31, 32, 44]. In order to evaluate the hypothesis that HAART may restrict the development of MS, Gold et al. investigated the RR of MS in their cohort of HIV-positive patients versus healthy controls 1 and 5 years after the initial patient admission upon HIV diagnosis [19]. This analysis revealed a time-dependent reduction in the noted RR of MS in the HIV-positive patients versus healthy controls from 0.38 (95% CI 0.15 to 0.79) overall, to 0.25 (95% CI 0.07 to 0.65, *p* < 0.005) after 1 year, and 0.15 (95% CI < 0.01 to 0.83, *p* = 0.04) after 5 years. Based on these findings of progressive decline of MS risk over time, the authors suggested that since most patients were started on HAART after HIV diagnosis and first admission in the study, the effect of HAART might have been the main determinant of the noted declining RR for MS over time [19]. These data, in line with the aforementioned reports of patients presenting improved outcomes of MS after HAART induction, have supported the view that HAART may have a possible protective effect in MS [7, 13, 31, 32, 44].

**Table 1:** Clinical characteristics of reported MS patients (with or without HIV infection) treated with antiretroviral medication

	Sex	Age at MS diagnosis	Age at HIV diagnosis	MS type	HIV treatment	Disease-modifying MS therapy	MS course	Follow-up (years)
(Chalkley & Berger, 2014) [7]	M	32	32	RRMS	tenofovir, emtricitabine, nelfinavir	none	NEDA	8
(Maruszak et al., 2011) [31]	M	26	Months before MS diagnosis	RRMS	combined treatment including nevirapine, stavudine, didanosine, lamivudine	none	Improvement of MS symptoms, no clinical relapses	2
(Maulucci et al., 2015) [32]	F	19	22	RRMS	tenofovir, emtricitabine, etravirine	Low-dose IFN beta-1a	NEDA-3	4
(Skarlis et al., 2017) [44]	M	24	36	RRMS	tenofovir-disorpoxil fumarate, emtricitabine, efavirenz	none	Annualized relapse rate of 0.28, EDSS progression 0.14	3
(Duran et al., 2004) [14]	M	32	32	RRMS	efavirenz, zidovudine, lamivudine	none	NEDA	1.5
(Drosu et al., 2018) [13]	F	25	No HIV	RRMS	zidovudine, lamivudine	none	NEDA-3	3

*Abbreviations*: *MS* Multiple sclerosis, *HIV* Human immunodeficiency virus, *M* Male, *F* Female, *RRMS* Relapsing-remitting MS, *INF* Interferon, *NEDA* No evidence of disease activity [18], *EDSS* Expanded disability status scale

A number of underlying pathophysiological mechanisms for the possible effects of antiretroviral drugs on MS have been proposed. Firstly, antiretroviral regimens have been suggested to act not only against HIV, but also on endogenous retroviruses [31, 34]. The expression of human endogenous retroviruses (HERVs) has been suggested as a possible predisposing factor for MS development [35]. Therefore, inhibition of HERVs by HAART, i.e., by inhibition of endogenous reverse transcriptase [31], may account for a protective effect of antiretroviral agents in MS. Secondly, chemical similarities between fumaric acid, which is contained in some combined antiretroviral regimens (e.g. the tenofovir disoproxil fumarate, which combined with efavirenz and emricitabine was reported in the treatment of a HIV-MS patient [44]) and the FDA-approved MS drug dimethylfumarate have been suggested to account for the noted MS stabilization in treated patients [7]. Nevertheless, it is disputable whether the amount of fumaric acid in tenofovir disoproxil fumarate or the biochemical similarities between fumaric acid and dimethylfumarate suffice to induce therapeutic effects in MS. Thirdly, given the established epidemiological link between MS and Epstein-Barr virus (EBV), a beneficial effect of nucleoside analogues mediated by inhibition of EBV DNA replication in MS patients has been hypothesized [13]. For example, zidovudine, a component of the HIV medication combivir, has been shown to inhibit effectively EBV [29], but has never been tested in randomized clinical trials for MS. Drosu et al. presented recently a case of a HIV-negative female patient with relapsing-remitting MS, whose MS both clinically and radiographically abated following institution of HAART with combivir [13]. Fourth, the effect of antiretroviral treatment on regulatory T cells (Treg) has been discussed as alternative explanation for the noted clinical stability of MS under antiretroviral treatment [41, 44]. Several studies have indicated that downregulation of Treg contributes to central nervous system injury in MS, which is then mediated by autoreactive CD4+ T lymphocytes, Th17 and Th1 cells [44]. Antiretroviral therapies may influence levels of Treg, CD4+, Th17 and Th1 cell populations, thereby suppressing the HIV-induced autoimmune diathesis [41].

Among the published cases of beneficial effects of antiretroviral therapy on MS activity (Table 1), the use of the following regimens has been reported: a) tenofovir, emtricitabine and etravirine [32], b) tenofovir-disorpoxil fumarate, emtricitabine and efavirenz [44], c) tenofovir, emtricitabine and nelfinavir [7], d) zidovudine and lamivudine [13], e) combined treatment including nevirapine, stavudine, didanosine and lamivudine [31], and f) efavirenz, zidovudine and lamivudine [14]. To date, no experimental evidence exists regarding the differential effects of antiretroviral agents on MS. Nevertheless, a recently published study, which investigated the in vitro effects of efavirenz (a non-nucleoside reverse transcriptase inhibitor) on the cellular expression of the envelope gene of MS-related human endogenous retrovirus (MSRV/HERV-Wenv) in healthy controls, demonstrated that efavirenz reduced MSRV/HERV-Wenv expression on lymphoblastoid cell lines [34]. As the MSRV/HERV-Wenv expression has been shown to be higher in MS patients compared to healthy controls [34, 35], the reduced expression of MSRV/HERV-W in vitro indicates a potential mechanism of action of antiretroviral medications in MS. Besides efavirenz, Morandi et al. further treated cells in vitro with lamivudine, tenofovir, daranuvir and raltegravir; yet, none of these drugs led to decreased expression of MSRV/HERV-Wenv [34]. Intriguingly, when cells were treated in vitro with combination of all aforementioned drugs (i.e., efavirenz, lamivudine, tenofovir, daranuvir and raltegravir), in an attempt to mimic the effects of combined HAART in vivo, a reduced expression of MSRV/HERV-Wenv RNA was noted. This finding indicates that drug synergy may be required to effectively reduce HERV-W expression and is in line with the well-established efficacy of HAART against HIV as opposed to single drug treatment [34].

To date, only one phase II clinical trial (INSPIRE) has investigated the effects of antiretroviral treatment in MS [20]. This trial studied the effect of the integrase inhibitor raltegravir in patients with relapsing-remitting MS, monitoring disease activity with monthly clinical and magnetic resonance imaging (MRI) follow-ups. Raltegravir was found to have no impact on MS disease activity [20]. Yet, the researchers discussed a number of reasons for the noted nil findings, including the choice of antiretroviral agent, the short treatment period (of 3 months) and the possible need for a combination of agents. In order to better delineate the possible effects on HAART in MS and determine appropriate antiretroviral drugs, drug combinations or optimal treatment periods, further experimental evidence from in vitro and animal model studies is warranted.

## Future research directions

Apart from antiretroviral HIV drugs, chemokine receptor inhibitors, such as the CCR5 antagonist maraviroc, which act as entry inhibitors of HIV-1, have been recently drawing growing attention with respect to HIV treatment [25]. Beyond HIV, the repurposing of CCR5 antagonists for the management of neuroinflammatory diseases, including MS, has also become a matter of current debate [30]. Several lines of evidence have indicated an elevated expression of CCR5 on T cells, macrophages and microglia in MS [2, 45, 48]. Also in MS patients with immune reconstitution syndrome (IRIS) and PML, the CCR5 antagonist maraviroc has been increasingly used to silence the overshooting immune response against the JC virus [38]. Furthermore, combined neutralization of all three known CCR5 ligands (CCL3, CCL4, CCL5) has been shown to ameliorate the course, i.e., reduce disease activity, in MS animal models of experimental autoimmune encephalomyelitis [43]. The question of whether beneficial effects may be noted after combination of CCR5 inhibitors with antiretroviral regimens for MS treatment warrants further research, as so far, no in vitro or animal model studies exist regarding efficacy of combined chemokine receptor inhibitors and antiretroviral drugs in MS. Finally, due to the established good tolerance and safety profile of modern HIV treatments [26], further in vitro and animal studies should be designed to guide optimal drug selection, dosing and drug combination for MS therapy.

## Conclusion

Although accumulating evidence in the literature suggests a negative association between HIV and MS in terms of a reduced risk of developing MS in HIV infected patients, further epidemiological studies are needed to corroborate these findings. In addition, observational evidence suggests a possibly reduced relapse rate in HIV patients with MS. Yet, it remains to date unclear whether this could be attributed to the HIV infection itself, HIV treatment or the combination of both. Concerning the primary use of antiretroviral or other HIV therapies in MS, the so-far existing experimental evidence is insufficient to substantiate the use of HIV therapies (including antiretroviral drugs and chemokine inhibitors) in MS patients without HIV infection. The negative findings of the recent phase II clinical trial (INSPIRE) indicate that further basic research is required to investigate appropriate antiretroviral drugs, drug combinations or optimal treatment periods for MS studies. In conclusion, as preliminary experimental evidence currently suggests that HIV treatments may hold therapeutic potential for MS, further studies are warranted to firstly, delineate the immunological mechanisms underlying possible efficacy of HIV drugs in MS, and to secondly, assess whether repurposing of HIV treatments for MS could be a worthwhile future research objective.

## Data Availability

Not applicable.

## Abbreviations

AIDS: Acquired immunodeficiency syndrome
CSF: Cerebrospinal fluid
EBV: Epstein-Barr virus
GBS: Guillain–Barré syndrome
HAART: Highly active antiretroviral therapy
HERVs: Human endogenous retroviruses
HIV: Human immunodeficiency virus
IRIS: Immune reconstitution syndrome
IRR: Incidence rate ratio
MG: Myasthenia gravis
MS: Multiple sclerosis
PML: Progressive multifocal leukoencephalopathy
RR: Rate ratio
Treg: Regulatory T cells
